# Association between atherosclerosis, hearing recovery, and hearing in the healthy ear in idiopathic sudden sensorineural hearing loss: a retrospective chart analysis

**DOI:** 10.1038/s41598-022-25593-5

**Published:** 2022-12-13

**Authors:** Nobuyoshi Tsuzuki, Koichiro Wasano, Naoki Oishi, Ko Hentona, Marie Shimanuki, Takanori Nishiyama, Yoshihiko Hiraga, Masafumi Ueno, Narihisa Suzuki, Seiichi Shinden, Kaoru Ogawa, Hiroyuki Ozawa

**Affiliations:** 1 Department of Otolaryngology, Head and Neck Surgery, Keio University School of Medicine, 35, Shinanomachi, Shinjuku, Tokyo 160-8582 Japan; 2 Department of Otolaryngology, Hiratsuka City Hospital, 1-19-1 Minamihara, Hiratsuka-City, Kanagawa 254-0065 Japan; 3 Department of Otolaryngology, Head and Neck Surgery, Tokai University School of Medicine, 143 Shimokasuya, Isehara-City, Kanagawa 259-1193 Japan; 4 National Institute of Sensory Organs, National Hospital Organization Tokyo Medical Center, 2-5-1 Higashigaoka, Meguro, Tokyo 152-8902 Japan; 5 Department of Otolaryngology, National Hospital Organization Tokyo Medical Center, 2-5-1 Higashigaoka, Meguro, Tokyo 152-8902 Japan; 6 Department of Otolaryngology, Saiseikai Utsunomiya Hospital, 911-1 Takebayashimachi, Utsunomiya-City, Tochigi 321-0974 Japan; 7 Department of Otolaryngology, Kawasaki Municipal Hospital, 12-1 Shinkawadori, Kawasaki, Kawasaki-City, Kanagawa 210-0013 Japan; 8 Department of Otolaryngology, Japanese Red Cross Shizuoka Hospital, 8-2 Outemachi, Aoi, Shizuoka-City, Shizuoka 420-0853 Japan

**Keywords:** Auditory system, Inner ear, Neurology, Risk factors

## Abstract

Atherosclerosis is reported to be a risk factor for the severity of idiopathic sudden sensorineural hearing loss (ISSNHL). We evaluated the hypothesis that atherosclerosis affects the hearing thresholds of both the affected and healthy sides of ISSNHL patients. We conducted multivariate analyses on retrospectively collected data of patients with ISSNHL (N = 762) to evaluate the relationship between known factors linked to atherosclerosis and hearing thresholds on affected and healthy sides and whether these factors are prognostic for hearing recovery. Older ages, vertigo or dizziness, diabetes mellitus, and congestive heart failure were significantly related to higher hearing thresholds on the affected side. Older ages, male, and vascular disease were significantly related to higher hearing thresholds on the healthy side. Vertigo or dizziness, severe hearing loss and hearing loss at high frequencies on the affected side, higher hearing thresholds on the healthy side, regular anticoagulant medication, and delayed steroid treatment were significantly related to lack of recovery. Since several atherosclerosis-related factors are associated with higher hearing thresholds on both affected and healthy sides in ISSNHL and higher hearing thresholds on the healthy side predict poorer prognosis, diagnosis, and predicting prognosis of ISSNHL may benefit from rigorous evaluation of patients’ cardiovascular comorbidities and hearing levels on both the healthy and affected sides.

## Introduction

The etiology of idiopathic sudden sensorineural hearing loss (ISSNHL) remains unknown, more than half of patients don’t completely recover^1^, and it continues to degrade the quality of life of many patients. The pathogenesis of ISSNHL also remains unclear, with some reports suggesting it may involve viral infections^2^, autoimmunity^3^, circulatory disturbances^4^, among others. Although the etiology and pathogenesis of ISSNHL have been studied for decades, major advances in these areas have been elusive. This gap has hindered diagnosis, selection of appropriate preventive measures, and testing and development of therapeutic strategies.

One reason for this lack of major progress may be that the diagnostic criteria for ISSNHL depend on the mode of onset and the form of the audiogram of the affected side^1^. Indeed, hearing thresholds on the patient’s unaffected side, or healthy side, are typically ignored. This constraint means that the hearing loss of ISSNHL is treated as a single disease, even though it arises from various pathologies. Supplemental Table 1 shows the criteria currently used for diagnosing ISSNHL published in 2015 by the Sudden Deafness Research Committee of the Ministry of Health, Labour and Welfare (MHLW) in Japan^1^, which is consistent with clinical practice guideline by American Academy of Otolaryngology-Head and Neck Surgery (AAO-HNS)^5^.

**Figure 1:**
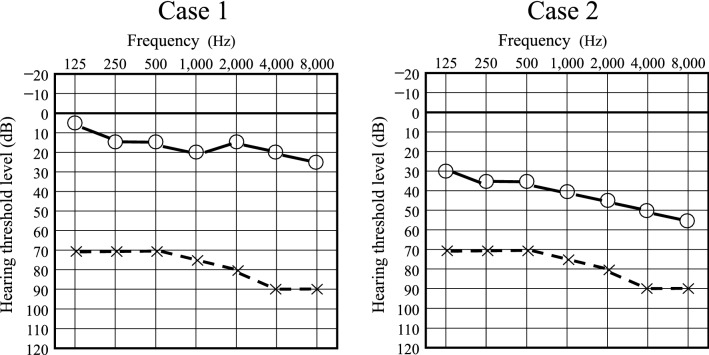
Two hypothetical cases showing two different patterns of idiopathic sudden sensorineural hearing loss (ISSNHL) classified as having similar severity using current diagnostic criteria. In Case 1, hearing threshold levels on healthy side are almost in the normal range. In Case 2, hearing threshold levels on healthy side are in the range of mild to moderate hearing loss. x symbols and dashed lines indicate threshold values on the left (affected) side; open circles and solid lines indicate threshold values on the right (healthy) side.

How do currently used criteria affect the evaluation of difficult-to-diagnose hearing disorders? Take, for example, the two hypothetical cases shown in Fig. 1. If these two cases were evaluated using only the audiogram of the affected side, the two cases might be classified as having similar severity using current criteria, although they are clearly different.

In the present study, we focused our study on audiograms of the healthy side in patients with ISSNHL. The pattern of the audiogram of the patient’s healthy side may reflect normal changes related not only to aging^6^, but also to a variety of medical conditions and factors related to a patient’s background, such as whether the patient has atherosclerosis and is subject to certain genetic factors^7,8^. Therefore, we hypothesized that the audiogram on the healthy side of patients with ISSNHL could be a useful additional measure to aid in the diagnosis and prognosis of ISSNHL, which is currently not done. With this approach, it may be possible to distinguish the two patterns of hearing loss shown in Fig. 1 of the two hypothetical ISSNHL patients, and then better direct treatment and understand a particular patient’s prognosis for recovery.

There are some suggestions in the literature that analyzing the audiogram on the healthy side in patients with ISSNHL may be beneficial. One report suggested that the pattern of hearing levels on the healthy side are predictive of a good/poor prognosis in older patients with ISSNHL^9^. Although the study had few subjects, older patients with normal hearing on the healthy side had a better chance for recovery than those with hearing loss on the healthy side. These findings and ideas prompted us to study the influence of various factors on hearing level not only on the affected side of ISSNHL patients but also on the healthy side, as well as to determine whether they might be prognostic factors.

We were particularly interested in evaluating the influence of known factors linked to atherosclerosis on ISSNHL occurrence and severity, because these factors are reported to also present a risk for triggering ISSNHL, and they can impact both severity of ISSNHL and the progression of age-related hearing loss^7,10–18^. We hypothesized that certain atherosclerosis factors affect hearing levels on both the affected and healthy sides in ISSNHL patients and have the potential to provide an additional measure to classify ISSNHL patients. Specifically, patients with ISSNHL who also have atherosclerosis, are more likely to be severely affected, and because atherosclerosis likely affects both sides equally, age-related hearing loss on the healthy side may be more advanced. Thus, in patients with ISSNHL who also have factors linked to the development of atherosclerosis, the severity on the affected side and elevated PTA thresholds on the healthy side may occur simultaneously.

The aim of the present study was to evaluate possible associations between particular patient variables—mainly atherosclerosis factors—and hearing levels (PTA) on both the affected and healthy sides in patients with ISSNHL. Multivariate analysis allowed us to determine the association between hearing levels on the healthy side and prognosis of ISSNHL (recovered/not recovered), controlling for a number of patient variables.

## Methods

### Patients and source of data

We conducted a retrospective study by analyzing data from medical charts of ISSNHL patients treated previously at six tertiary-level hospitals in Japan. These study subjects and some of their data were described fully in our previous report^19^. For the present study, we used the same dataset but excluded patients who had vestibular schwannoma, which was verified by head MRI. We also included additional data from the included patients’ charts for analysis in the present study. Any patients diagnosed with bilateral simultaneous onset of ISSNHL were also excluded.

Briefly, the original dataset^19^ comprised audiometric, demographic, and basic medical data gathered from the medical records of 916 SSNHL patients that sought treatment between 2012 and 2020. For the included patients in the present study, we analyzed additional data from their charts about hearing prognosis (recovery/no recovery) and audiometric data from their healthy side. The diagnosis of ISSNHL was done using the criteria of the Sudden Deafness Research Committee of the Ministry of Health, Labour and Welfare (MHLW), Japan (2015) (Supplemental Table 1)^1^. The criteria for ISSNHL are the Japanese guideline which is almost consistent with clinical practice guideline by AAO-HNS^5^.

### Chart data collected for analysis

We obtained data from the patients’ charts about their age, sex, affected side regarding ISSNHL (left ear [L] or right ear [R]), pure tone audiogram (PTA) on the affected side assessed at standard testing frequencies (below), presence of vertigo or dizziness (V/D) symptoms, and selected comorbidities^19^. The latter included diabetes mellitus (DM), hypertension (HTN), hyperlipidemia (HLD), atrial fibrillation (Afib), stroke or transient ischemic attack (TIA), venous thromboembolism (VTE), congestive heart failure (CHF), and any indicators of vascular disease (e.g., myocardial infarction, angina pectoris, peripheral artery disease, aortic plaque)^19^. Additional data we collected from the patients’ charts for the present study were current or past smoking status (yes/no); hearing thresholds (dB; decibel hearing level) at low frequencies (125, 250, 500 Hz); hearing thresholds (dB) at middle frequencies (500, 1000, 2000 Hz); hearing thresholds (dB) at high frequencies (2000, 4000, 8000 Hz); PTA on the healthy side; regular antiplatelet medication at onset of hearing loss (yes/no); regular anticoagulant medication at onset of hearing loss (yes/no), systemic steroid therapy, initial or salvage intratympanic steroid therapy; latency (days) to begin steroid treatment, prostaglandin E1 treatment, and whether they received hyperbaric oxygen therapy. We also gathered from the patients’ charts whether they recovered (yes/no) from their hearing loss related to ISSNHL. Some patients never received steroid treatment, systemic steroid therapy, or intratympanic steroid therapy. For those patients, they were manually assigned to the group who began steroid treatment 8 or more days after ISSNHL onset.

To get a clearer picture of the patients’ hearing level, we calculated the arithmetic mean of individual PTA data obtained at five frequencies (250, 500, 1000, 2000, 4000 Hz) as follows: mean PTA = (PTA_250_ + PTA_500_ + PTA_1000_ + PTA_2000_ + PTA_4000_)/5. The severity of hearing loss on the affected side was graded according to the following MHLW criteria, the Japanese guidelines for ISSNHL: Grade 1: PTA < 40 dB; Grade 2: 40 dB ≤ PTA < 60 dB; Grade 3: 60 dB ≤ PTA < 90 dB; and Grade 4: 90 dB ≤ PTA^1^. The severity of hearing loss on the healthy side was graded according to the following criteria: Grade 0: PTA < 25 dB; Grade 1: 25 dB ≤ PTA < 40 dB; Grade 2: 40 dB ≤ PTA < 60 dB; Grade 3: 60 dB ≤ PTA < 90 dB; and Grade 4: 90 dB ≤ PTA. Hearing loss at low, middle, and high frequencies was also estimated. Hearing loss at low, middle, and high frequencies was defined as a hearing loss of ≥ 30 dB at two or more of the three frequencies (e.g., at low frequencies, two or more of 125, 250, 500 Hz).

The first PTA obtained post-ISSNHL onset that had complete data was used for analysis. Patients lacking sufficient description in their medical charts were excluded in order to have a complete-case analysis.

### Analysis strategy for assessing association between atherosclerosis factors and hearing levels on affected and healthy side in ISSNHL

To evaluate possible associations between previously studied factors known to be linked to atherosclerosis and hearing levels on each side (affected and healthy) in patients with ISSNHL, we conducted multivariate regression analyses. Hearing levels on affected side and healthy side (dB thresholds) were analyzed in separate models. We carried out a three-step analysis. First, we selected independent variables related to hearing levels on each side based on previous literature. The selected independent variables on affected side included potential factors related to the severity of ISSNHL: patient age, DM, HTN, HLD, current or past smoking status; cardiac disease (e.g., CHF, vascular disease, Afib); and presence of V/D symptoms^10–13^. For evaluation of the healthy side, the independent variables included potential risk factors for age-related hearing loss: patient age, sex, DM, HTN, HLD, current or past smoking status, cardiac disease, and stroke or TIA^14–18,20,21^.

Second, we performed separate univariate regression analyses for the affected and healthy sides. The single outcome we were interested in for each side was what was the percentage of variance (expressed as r^2^) in PTA (dependent variable) explained by certain clinical characteristics (independent variables). The independent variables were patient age, sex, affected ear (L/R), presence of V/D symptoms, current or past smoking status, and patient comorbidities (DM, HTN, HLD, Afib, stroke or TIA, VTE, CHF, and vascular disease). If a variable had a significant P-value of less than 0.20 for the outcome, it was included in the final multivariate model.

For the third step in our analysis, the final multivariate model was constructed by fitting multiple linear regression models that evaluated the relationship between PTA on the affected or healthy side (dependent variable) and the various independent variables. The independent variables selected in the first and second steps were incorporated into the final model. We performed normal log-transformation on the dependent variable (PTA) when residuals in the model were non-normal.

### Analysis strategy for assessing association between hearing levels on the healthy side and prognosis for recovery

To determine possible associations between hearing levels on the patients’ healthy side and prognosis for recovering from ISSNHL, we carried out a two-step statistical analysis. First, we used Fisher’s exact test to evaluate the relationship between recovery of hearing loss (yes/no) and other relevant data we collected. Recovery was defined as a study subject’s audiogram having one of the following two patterns: (1) the arithmetic mean of thresholds obtained at five frequencies (250, 500, 1000, 2000, and 4000 Hz) on the affected side was 20 dB or less; (2) If there was hearing loss on the healthy side and the hearing level on the healthy side was determined to be stable, the arithmetic mean of thresholds at five frequencies (250, 500, 1000, 2000, and 4000 Hz) on the affected side recovered to within 10 dB of the healthy side’s arithmetic mean of the thresholds at the five frequencies. These definitions were determined with reference to the Japanese and AAO-HNS guidelines which described the definition of recovery using the hearing level on the healthy side^1,22^.

For the second step of our analysis of prognostic factors, we conducted a multivariate regression analysis. The final multivariate model was developed by fitting a multivariate logistic regression model using recovery/no recovery of hearing loss as the dependent variable and the independent variables for factors previously reported as prognostic factors reported for ISSNHL recovery^9,23–37^. These variables were patient age, DM, HTN, HLD; use of systemic steroid therapy, intratympanic steroid therapy (initial or salvage), latency to begin steroid treatment, use of prostaglandin E1, use of hyperbaric oxygen therapy; severity of hearing loss on the affected side, pattern of audiogram (i.e., presence of hearing loss at low, middle, high frequencies), severity of hearing loss on the healthy side; and presence of V/D symptoms. We also included as independent variables all variables from the univariate analyses that had a P-value of less than 0.20.

The cutoff values for MHLW Grade level of the affected side and Grade level of the healthy side were determined appropriately based on the distribution of Grades. The cutoff values for patient age and latency (days) to begin steroid treatment were based on values reported in previous studies^28–30^.

Since some patients lacked complete data and were excluded from the complete-case analysis, we conducted a sensitivity analysis to evaluate the stability of the prognosis results for recovering from ISSNHL. One straightforward approach for dealing with missing data is to analyze only cases that have no missing data. However, this method can induce bias. Inverse probability weighting (IPW) is a commonly used method to correct this bias. We used IPW to evaluate selection bias due to missing outcome data (recovery/no recovery from ISSNHL)^38^. Using a dataset that excluded patients with any missing baseline characteristics data, we calculated the probability of missing values in the outcome variable using logistic regression that included variables in baseline characteristics. We used an IPW-weighted multivariate logistic regression model to determine the ISSNHL prognostic factors and to compute odds ratios for the lack of ISSNHL recovery outcome.

### Statistical analysis

For all analyses, we used EZR (Easy R; version 1.54) statistical software^39^. EZR is based on R and R commander and is available at http://www.jichi.ac.jp/saitama-sct/SaitamaHP.files/statmed.html. Population means, standard deviations (SD), and counts were also calculated with EZR. For all tests, a P-value of < 0.05 was defined as statistically significant. For multivariate analyses, we used the variance inflation factor (VIF) to check for the presence of multicollinearity in the independent variables. A VIF cutoff of less than 5 was adopted. The VIF reflects the degree to which linear dependencies among the independent variables is inflated due to collinearity. It is generally accepted that a VIF value > 10 is unacceptable for multiple regression^40^.

### Ethics statement

As the current study used the same dataset as that reported in our previous study^19^, details of institutional approvals are described in that publication^19^. Briefly, the typical requirement to obtain written informed consent was waived by the institutional review boards because of the retrospective design. Participant data were anonymized, and all efforts were made to protect participant data. We carried out the present study in accordance with the guidelines of the Strengthening the Reporting of Observational Studies in Epidemiology (STROBE) statement^41^.

## Results

### Study participant characteristics

**Figure 2:**
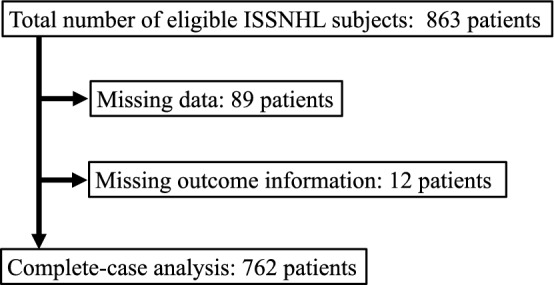
Flow diagram for study inclusion. ISSNHL, idiopathic sudden sensorineural hearing loss.

A total of 863 patients with ISSNHL met the inclusion criteria. However, 101 of the 863 patients had missing data and were excluded from the complete-case analysis (Fig. 2). Thus, 762 patients (351 male, 411 female) contributed data to the complete-case analysis for the study. Table 1 shows demographic and clinical characteristics of these included participants.

**Table 1:** Characteristics of study participants with ISSNHL.

Characteristic	Count (%)^a^
N	762
Mean age, years (SD)	59.8 (15.1)
**Sex**	
Male	351 (46.1)
Female	411 (53.9)
**Affected ear**	
Left	396 (52.0)
Right	366 (48.0)
Current or past smoking, yes	236 (31.0)
Diabetes mellitus (DM)	131 (17.2)
Hypertension (HTN)	222 (29.1)
Hyperlipidemia (HLD)	182 (23.9)
Vascular disease	59 (7.7)
Venous thromboembolism (VTE)	8 (1.0)
Congestive heart failure (CHF)	17 (2.2)
Atrial fibrillation (Afib)	17 (2.2)
Stroke or transient ischemic attack (TIA)	27 (3.5)
Regular anticoagulant medication at onset of hearing loss, yes	28 (3.7)
Regular antiplatelet medication at onset of hearing loss, yes	74 (9.7)
Vertigo/Dizziness	225 (29.5)
PTA (dB) on affected side, mean (SD)	61.8 (23.0)
**Initial MHLW**^**b**^** Grade of hearing loss, affected ear**	
1	151 (19.8)
2	226 (29.7)
3	278 (36.5)
4	107 (14.0)
**Hearing loss**	
Low frequencies	653 (85.7)
Middle frequencies	717 (94.1)
High frequencies	678 (89.0)
PTA (dB) on healthy ear, mean (SD)	24.7 (17.9)
**Initial Grade of hearing loss, healthy ear**	
0	483 (63.4)
1	187 (24.5)
2	53 (7.0)
3	23 (3.0)
4	16 (2.1)
Received systemic steroid therapy, yes	715 (93.8)
Received intratympanic steroid therapy (initial or salvage), yes	40 (5.2)
**Latency to begin steroid treatment**^**c**^	
0–7 days post-onset	602 (79.0)
8 or more days post-onset	160 (21.0)
Received prostaglandin E1 treatment, yes	131 (17.2)
Received hyperbaric oxygen therapy, yes	12 (1.6)
**Hearing prognosis (outcome)**	
Recovered	280 (36.7)
Failed to recover	482 (63.3)

SD, standard deviation; PTA, pure tone audiogram; PTA values represent the arithmetic mean of hearing threshold (measured in dB) obtained at 5 test frequencies (250, 500, 1000, 2000, 4000 Hz).

^a^Unless otherwise indicated, values are counts (percentage); N represents patients who had unilateral ISSNHL and who had no evidence of vestibular schwannoma.

^b^Criteria of the Sudden Deafness Research Committee of the Ministry of Health, Labour and Welfare.

^c^Some patients never received steroid treatment; they were manually assigned to the 8-or-more-days group.

### Associations between known factors linked to atherosclerosis and hearing levels on affected and healthy sides in ISSNHL

Several factors previously linked to atherosclerosis predicted severity of hearing loss (higher PTA) in patients diagnosed with ISSNHL. Table 2 shows separately the results of the univariate regression analyses for evaluating atherosclerosis factors and their possible association with PTA for the affected and healthy sides of participants with ISSNHL. The variable accounting for the most variance in PTA on the healthy side was patient age (r^2^ = 0.145), while on the affected side, V/D symptoms accounted for the most variance (r^2^ = 0.050). For both the affected and healthy side, PTA was significantly associated with patient age, presence of V/D symptoms, DM, HTN, and vascular disease. Other factors linked with atherosclerosis showed different patterns in their association with PTA on the affected and healthy sides in patients with ISSNHL. Stroke or TIA, CHF, and VTE were significantly associated with PTA only on the affected side. Sex of the patient and Afib were significantly associated with the PTA only on the healthy side. HLD and Afib were the only factors in our univariate model that were not significantly associated with the PTA on the affected side, which previously had been reported to be closely associated with the occurrence and severity of ISSNHL^11,12^.

**Table 2:** Results of univariate regression analysis of atherosclerosis factors and hearing loss (PTA).

	PTA on affected side^a^					PTA on healthy side^a^				
	Coefficient	SE	95% CI	P-value	r^2^	Coefficient	SE	95% CI	P-value	r^2^
Age	0.183	0.055	0.075 to 0.290	< 0.01***	0.014	0.451	0.040	0.373 to 0.529	< 0.01***	0.145
Sex (male = 0, female = 1)	− 1.275	1.675	− 4.562 to 2.012	0.45	0.001	− 3.000	1.298	− 5.549 to − 0.452	0.02*	0.007
Current or past smoking	0.761	1.806	− 2.783 to 4.306	0.67	0.000	2.009	1.403	− 0.744 to 4.763	0.15	0.003
Affected ear (left = 0, right = 1)	3.510	1.666	0.239 to 6.782	0.04*	0.006	− 1.305	1.299	− 3.855 to 1.245	0.32	0.001
Vertigo/dizziness (V/D)	11.223	1.785	7.720 to 14.727	< 0.01***	0.050	− 2.931	1.420	− 5.718 to − 0.144	0.04*	0.006
Diabetes mellitus (DM)	7.521	2.196	3.210 to 11.832	< 0.01***	0.015	4.638	1.713	1.275 to 8.001	< 0.01**	0.010
Hypertension (HTN)	3.947	1.832	0.350 to 7.543	0.03*	0.006	7.440	1.404	4.684 to 10.195	< 0.01***	0.036
Stroke or transient ischemic attack (TIA)	9.943	4.502	1.105 to 18.781	0.03*	0.006	3.155	3.511	− 3.738 to 10.047	0.37	0.001
Congestive heart failure (CHF)	21.584	5.599	10.592 to 32.576	< 0.01***	0.019	5.566	4.393	− 3.058 to 14.189	0.21	0.002
Vascular disease	12.015	3.094	5.942 to 18.088	< 0.01***	0.019	11.917	2.391	7.223 to 16.611	< 0.01***	0.032
Venous thromboembolism (VTE)	16.519	8.170	0.480 to 32.558	0.04*	0.005	10.248	6.361	− 2.240 to 22.735	0.11	0.003
Hyperlipidemia (HLD)	2.051	1.957	− 1.791 to 5.893	0.30	0.001	2.279	1.521	− 0.707 to 5.264	0.13	0.003
Atrial fibrillation (Afib)	8.649	5.645	− 2.433 to 19.731	0.13	0.003	14.350	4.366	5.778 to 22.922	< 0.01**	0.014

Coefficient, regression coefficient; CI, confidence interval; SE, standard error of the regression coefficient; other abbreviations same as in Table 1.

*p < 0.05, ** p < 0.01, ***p < 0.001.

^a^PTA values represent the arithmetic mean of hearing threshold (measured in dB) obtained at 5 test frequencies (250, 500, 1000, 2000, 4000 Hz).

Table 3 shows results of the multivariate regression analysis predicting PTA on the affected side from atherosclerosis factors previously reported to be associated with the severity of ISSNHL. Several of the reliably associated variables identified in the univariate analysis were no longer significant in the multivariate regression model. Older age of patient, presence of V/D symptoms, DM, and CHF were the only factors significantly associated with a higher threshold of PTA on the patients’ affected side.

**Table 3:** Results of multivariate regression of atherosclerosis factors predicting severity of hearing loss (PTA) on affected side.

Independent variable	PTA on affected side			
	Coefficient	SE	95% CI	p-value
(Intercept)	45.419	3.592		
Age	0.155	0.058	0.042 to 0.269	< 0.01**
Current or past smoking	0.050	1.767	− 3.418 to 3.519	0.98
Affected ear (left = 0; right = 1)	3.152	1.600	0.012 to 6.293	< 0.05*
V/D	12.210	1.777	8.722 to 15.698	< 0.01***
DM	5.907	2.233	1.523 to 10.291	< 0.01**
HTN	0.824	1.930	− 2.964 to 4.614	0.62
Stroke or TIA	2.920	4.603	− 6.117 to 11.958	0.53
CHF	13.335	5.841	1.869 to 24.801	0.02*
Vascular disease	5.425	3.356	− 1.164 to 12.013	0.11
VTE	8.228	8.374	− 8.211 to 24.666	0.33
HLD	− 0.986	1.984	− 4.881 to 2.910	0.62
Afib	0.707	5.623	− 10.331 to 11.745	0.90

Same abbreviations as in Table 1.

Dependent variable = PTA on affected side.

Multiple R^2^ for final model = 0.1092; adjusted R^2^ = 0.09491; p < 0.00001.

*p < 0.05, ** p < 0.01, ***p < 0.001.

Table 4 shows results of the multivariate regression analyses of PTA on the healthy side and the atherosclerosis factors. In the final model, PTA was normal log-transformed. The pattern of results for the healthy side was very different from the multivariate model for the affected side. Older age of patient, being male, and presence of vascular disease were the only significantly associated atherosclerosis factors predicting higher PTA thresholds on the patients’ healthy side.

**Table 4:** Results of multivariate regression of atherosclerosis factors predicting severity of hearing loss (PTA) on healthy side.

Independent variable	PTA on healthy side			
	Coefficient	SE	95% CI	p-value
(Intercept)	0.805	0.036		
Age	0.009	0.001	0.008 to 0.010	< 0.01***
Sex (Male = 0, Female = 1)	− 0.053	0.018	− 0.088 to − 0.018	< 0.01**
Current or past smoking	0.007	0.019	− 0.030 to 0.044	0.71
V/D	− 0.005	0.018	− 0.040 to 0.030	0.78
DM	0.012	0.022	− 0.032 to 0.056	0.58
HTN	0.032	0.019	− 0.006 to 0.070	0.10
Stroke or TIA	− 0.003	0.046	− 0.093 to 0.087	0.95
CHF	− 0.058	0.058	− 0.172 to 0.057	0.32
Vascular disease	0.068	0.033	0.003 to 0.134	0.04*
VTE	0.044	0.084	− 0.121 to 0.208	0.60
HLD	− 0.029	0.020	− 0.068 to 0.010	0.14
Afib	0.089	0.056	− 0.021 to 0.199	0.11

Same abbreviations as in Table 1.

Dependent variable = PTA on healthy side.

Multiple R^2^ for final model = 0.3078; adjusted R^2^ = 0.2967; p < 0.00001.

*p < 0.05, ** p < 0.01, ***p < 0.001.

### Hearing level of the healthy side was associated with ISSNHL prognosis

Supplemental Table 2 shows the results of the univariate analyses evaluating the relationship between recovery from ISSNHL and the relevant demographic and clinical variables. The recovery rate for each Grade level on the affected side was 53.0% for Grade 1, 37.6% for Grade 2, 36.3% for Grade 3, and 13.1% for Grade 4. The recovery rate for each Grade level on the healthy side was 37.5% for Grade 0, 41.7% for Grade 1, 28.3% for Grade 2, 17.4% for Grade 3, and 12.5% for Grade 4. Therefore, we determined that each of the cutoff values for Grade level of the affected and healthy sides were at Grade 4 and Grade 2, respectively, as reasonable cutoff based on the distribution of recovery rates for each Grade. Table 5 shows the results of the multivariate logistic regression model for predicting recovery from ISSNHL. Since all patients who received hyperbaric oxygen therapy did not recover from ISSNHL, this independent variable was excluded from the final multivariate logistic regression model. The following clinical characteristics significantly predicted a lack of recovery: presence of V/D symptoms, an initial hearing loss in the affected ear graded MHLW Grade 4, an initial hearing loss in the healthy ear graded Grade 2 or higher, higher PTA thresholds at high frequencies in the affected ear, regular anticoagulant medication at onset of hearing loss, and a latency to begin steroid treatment of 8 or more days. Only HTN was significantly associated with recovery from ISSNHL.

**Table 5:** Multivariate logistic regression model for predicting recovery from ISSNHL.

Independent variable	Adjusted OR	95% CI	p-value
**Age**			
< 60	1	(Reference)	
≥ 60	1.32	0.92 to 1.88	0.13
DM	0.83	0.53 to 1.29	0.41
HTN	0.60	0.41 to 0.87	< 0.01**
HLD	0.83	0.56 to 1.24	0.37
V/D	2.54	1.72 to 3.75	< 0.01***
**Initial MHLW**^**a**^** Grade of hearing loss, affected ear**			
1–3	1	(Reference)	
4	3.42	1.84 to 6.35	< 0.01***
**Initial Grade of hearing loss, healthy ear**			
0–1	1	(Reference)	
2–4	1.89	1.09 to 3.29	0.02*
**Hearing loss**			
Low frequencies	1.35	0.84 to 2.19	0.22
Middle frequencies	1.53	0.76 to 3.09	0.23
High frequencies	1.86	1.13 to 3.06	0.01*
Regular anticoagulant medication at onset of hearing loss	3.37	1.17 to 9.73	0.02*
Received systemic steroid therapy, yes	1.24	0.56 to 2.74	0.60
Received intratympanic steroid therapy (initial or salvage), yes	0.70	0.34 to 1.41	0.32
**Latency to begin steroid treatment**^**c**^			
0–7 days post-onset	1	(Reference)	
8 or more days post-onset	2.85	1.77 to 4.59	< 0.01***
Received prostaglandin E1 treatment, yes	0.83	0.54 to 1.27	0.38

Same abbreviations as in Table 1.

Dependent variable = no recovery.

OR, odds ratio; CI, confidence interval.

*p < 0.05, ** p < 0.01, ***p < 0.001.

^a^Criteria of the Sudden Deafness Research Committee of the Ministry of Health, Labour and Welfare.

Supplemental Table 3 shows the results of the sensitivity analysis using an IPW-weighted multivariate logistic regression model. This model was nearly identical to those of the complete-case analysis shown in Table 5.

## Discussion

The pathogenesis of ISSNHL is unknown, although circulatory disturbances^4^, intralabyrinthine hemorrhage^42^, viral infection^2^, and autoimmunity^3^ are reported to contribute to this frightening condition. The diagnostic criteria for ISSNHL are constructed based on the sudden onset of hearing loss and the hearing pattern on the affected side, as revealed in the patient’s PTA. These currently used criteria make it difficult to clearly identify the pathogenesis of ISSNHL, as a variety of pathologies may lead to ISSNHL. This deficiency, in turn, makes it difficult to develop an appropriate treatment.

Focusing on the pathogenesis of circulatory disturbances, atherosclerosis factors that are reported to be associated with ISSNHL include DM, HTN, dyslipidemia (DLP), and cardiovascular disease (CVD)^43,44^. Aging, DM, smoking, and a history of heart disease are reported to be factors in the severity of ISSNHL^10–13^. Aging, DM, and smoking are also factors in the development of atherosclerosis, which is in itself a risk factor for the development and severity of heart disease^45^.

In the present study, we evaluated factors related to circulatory disturbances and whether they predict the severity and prognosis of ISSNHL. Specifically, we focused on factors linked to the development of atherosclerosis and whether these are associated with hearing thresholds on the healthy side of patients with ISSNHL. These factors are not currently included in the diagnostic criteria for ISSNHL. We also examined whether factors linked to atherosclerosis are predictive of ISSNHL recovery. Specifically, we focused on the relationship between hearing thresholds on the affected and healthy sides in ISSNHL and prognostic factors for ISSNHL recovery.

We had two main findings. First, we documented significant associations between atherosclerosis factors and higher thresholds of PTA on the patients’ healthy side as well as on their affected side. Our study provided evidence that older ages, V/D, DM, and CHF are associated with higher thresholds of PTA on the patients’ affected side. Previous studies have reported that V/D, cardiac disease, atherosclerosis factors such as older age and DM are related to the severity of ISSNHL^10–12^.

We also found evidence that the ISSNHL patients’ healthy sides also show impairments in hearing. Although there are no reports on risk factors associated with an increase in hearing thresholds on the healthy side of patients with ISSNHL, the primary risk factors are those related to aging. There are a number of reported risk factors for age-related hearing loss, including advancing age, being male, smoking, alcohol use, DM, HTN, heart disease, noise exposure, and genetic predisposition^7,14–18,20,21,46^. In the present study, older ages, being male, and vascular disease were associated with higher PTA thresholds on the healthy side. To the best of our knowledge, there have been no reports examining the patient factors that influence hearing in the healthy ear in patients diagnosed with ISSNHL.

Our second main finding related to factors that affect the chances for hearing recovery in ISSNHL patients. Factors linked to atherosclerosis are reported to be prognostic factors for whether patients recover from ISSNHL. Specifically, the comorbidities of DM, HTN, DLP, metabolic syndrome, and Framingham CVD risk score lower the rate of ISSNHL recovery and lead to poorer prognosis^23–27^. In addition to factors linked to atherosclerosis, other medical conditions and hearing parameters are reported to be associated with ISSNHL. The severity of hearing loss on the affected side (hearing thresholds at the initial diagnosis of ISSNHL), advanced age, V/D, a flat or “down-sloping” audiogram, and latency from onset of hearing loss to start of treatment appear to be associated with prognosis and recovery rate of ISSNHL^9,28–30^. There are also reports of positive therapeutic effects of various treatments. For example, systemic steroid therapy, intratympanic steroid therapy (initial or salvage), prostaglandin E1 administration, and hyperbaric oxygen therapy are reported to have beneficial effects on ISSNHL, but none of these have been sufficiently evaluated in terms of how they impact recovery from ISSNHL^31–37^. In the present study, poor prognostic factors include presence of V/D symptoms, having an initial MHLW Grade 4 hearing loss in the affected ear, higher PTA thresholds at high frequencies in the affected ear, delaying steroid treatment 8 or more days after onset of hearing loss, initial Grade 2 or higher hearing loss in the healthy ear, and regular anticoagulant medication at onset of hearing loss. Some of these negative factors have been reported in the past. V/D, severe hearing loss in the affected ear, and delayed steroid treatment are reported to be poor prognostic factors for ISSNHL^25,28–30^. Higher PTA thresholds at high frequencies in the affected ear might have the same significance as that of flat or “downsloping” hearing audiograms, which have been reported to be poor prognostic factors^28^. To the best of our knowledge, there have been no reports indicating that higher PTA thresholds on the healthy side and regular anticoagulant medication at onset of hearing loss are poor prognostic factors for ISSNHL. However, the relationship between PTA on the healthy side and prognosis of ISSNHL, as well as the relationship between atherosclerosis factors and PTA on the healthy side, require further investigation. Although regular anticoagulant medication at onset of hearing loss might be a risk factor for intralabyrinthine infarction or hemorrhage, more rigorous investigation is needed, one that combines imaging of the inner ear and audiometric assessment in patients with ISSNHL.

Contrary to our expectations, HTN in the present study was a favorable prognostic factor for ISSNHL. Since the HTN variable in the present study was retrospective data gathered through chart review, we know that most of the study patients were treated properly with an antihypertensive drug. Therefore, lacking data about accurate blood pressure measurements at the point of ISSNHL development may have confounded our results.

The present study has two strengths. Because we were able to analyze data from a relatively large number of patients from six different hospitals, we were able to confidently carry out multivariate regression analyses. This first strength allowed us to comprehensively examine PTA on both the affected and healthy sides of patients with ISSNHL and rigorously evaluate several factors linked to atherosclerosis that may be predictors of ISSNHL and prognostic factors for recovery. The second strength was that we evaluated not only prognostic factors’ possible relationship to PTA on the affected side but also to PTA on the healthy side, which has not been reported as a prognostic factor for ISSNHL. With further study, our results might point to new advice about predicting prognosis of ISSNHL by assessing PTA on the healthy side.

The present study also has three possible limitations. First, the study may be influenced by selection bias related to the data coming from retrospective chart review. However, there were few missing outcome values in the prognostic factors analysis, and the results of IPW-weighted analysis were similar to those of the complete-case analysis. Moreover, the cases were consecutive cases arriving at the six hospitals. Therefore, we place high confidence in the reliability of the results.

Another cautionary note is that we mainly examined known factors linked to atherosclerosis in the analysis of hearing on the healthy side. Therefore, other factors that are known to affect hearing on the healthy side, such as noise exposure, alcohol consumption, and genetic predisposition, were not investigated.

Third, only a small number of patients received salvage treatment such as hyperbaric oxygen therapy or intratympanic steroid therapy in the present study. Therefore, insufficient adjustment for salvage treatment might have affected the analysis of hearing recovery. In Japan, intratympanic steroid therapy is rarely selected due to medical insurance coverage. In addition, hyperbaric oxygen therapy is not common in Japan because few medical facilities have the equipment for hyperbaric oxygen therapy.

In conclusion, certain atherosclerosis factors appear to be associated with higher PTA hearing thresholds not only on the patients’ affected side in ISSNHL but also with higher hearing thresholds on their healthy side. Higher hearing thresholds of 40 dB or higher on the healthy side were associated with poor prognosis for recovery from ISSNHL. Thus, a clearer picture of a patient’s ISSNHL onset and chances for recovery may emerge from a more rigorous evaluation of hearing levels on the unaffected, or healthy side, in addition to that done on the affected side.

In the near future, we will conduct prospective studies of ISSNHL and the impact of atherosclerosis factors, focusing on hearing on the healthy side. In addition, it will be informative to determine prospectively whether intralabyrinthine infarction or hemorrhage is present on MRI in patients with ISSNHL who were taking regular anticoagulant medication at the time of sudden hearing loss onset.

## Supplementary Information

Supplementary Tables.

## Data Availability

The dataset we used in the study of the present report partially overlaps with a previously published paper, excluding patients with vestibular schwannoma^19^. The analyzed data are available from the corresponding author upon reasonable request.
