# Supplementary material for: Association between atherosclerosis, hearing recovery, and hearing in the healthy ear in idiopathic sudden sensorineural hearing loss: a retrospective chart analysis
Source: Sci Rep. 2022 Dec 13;12:21571. doi: 10.1038/s41598-022-25593-5 (PMC9747959; doi:10.1038/s41598-022-25593-5)
Supplement: Supplementary file 1 — Supplementary Tables. [file 41598_2022_25593_MOESM1_ESM.pdf]

Supplemental Table 1 Diagnostic criteria* for ISSNHL in Japan

Main symptoms

- Sudden onset
- Severe sensorineural hearing loss
- Unknown etiology

For reference

- Hearing loss (i.e., decrement of 30 dB or more over three consecutive frequencies within 72 h)
    - Exclude cases diagnosed as acute low-tone sensorineural hearing loss
    - Exclude functional hearing loss
    - Sudden onset of hearing loss; may progressively deteriorate over a few days
    - No repeated episodes of improvement or worsening of hearing loss
    - Unilateral hearing loss, but may be bilateral at the onset
- May be accompanied by tinnitus around the time of onset of hearing loss
- May be accompanied by vertigo, nausea, and/or vomiting around the time of onset of hearing loss, without recurrent episodes of vertigo
- No cranial nerve symptoms, other than from cranial nerve VIII

Definitive diagnosis: All of the above main symptoms are present

*Criteria established by Research Committee of the Ministry of Health, Labour, and Welfare of Japan in 2015. Table adapted from reference 1.

Supplemental Table 2 Univariate analyses of risk factors related to prognosis of idiopathic sudden sensorineural hearing loss (ISSNHL)

Characteristic	Recovery (%)	No recovery (%)	Unadjusted OR	95% CI	P-value
N	280	482			
Mean age, years (SD)					
< 60	136 (40.7)	198 (59.3)	1	(Reference)	
≥ 60	144 (33.6)	284 (66.4)	1.35	1.00-1.84	<0.05*
Sex					
Male	127 (36.2)	224 (63.8)	1	(Reference)	
Female	153 (37.2)	258 (62.8)	0.96	0.70-1.30	0.82
Affected ear					
Left	144 (36.4)	252 (63.6)	1	(Reference)	
Right	136 (37.2)	230 (62.8)	0.97	0.71-1.31	0.82
Current or past smoking	91 (38.6)	145 (61.4)	0.89	0.64-1.24	0.51
Diabetes mellitus	50 (38.2)	81 (61.8)	0.93	0.62-1.40	0.77
Hypertension	92 (41.4)	130 (58.6)	0.75	0.54-1.05	0.10
Hyperlipidemia	75 (41.2)	107 (58.8)	0.78	0.54-1.11	0.16
Vascular disease	20 (33.9)	39 (66.1)	1.14	0.64-2.12	0.68
Venous thromboembolism	2 (25.0)	6 (75.0)	1.75	0.31-17.86	0.71
Congestive heart failure	6 (35.3)	11 (64.7)	1.07	0.36-3.55	1

Atrial fibrillation	5 (29.4)	12 (70.6)	1.40	0.45-5.14	0.62
Stroke or TIA	11 (40.7)	16 (59.3)	0.84	0.36-2.03	0.69
Regular anticoagulant medication at onset of hearing loss	5 (17.9)	23 (82.1)	2.75	1.01-9.38	0.04*
Regular antiplatelet medication at onset of hearing loss	28 (37.8)	46 (62.2)	0.94	0.56-1.62	0.90
Vertigo/Dizziness	50 (22.2)	175 (77.8)	2.62	1.81-3.83	<0.01***
Initial MHLW Grade of hearing loss, affected ear					
1	80 (53.0)	71 (47.0)			
2	85 (37.6)	141 (62.4)			
3	101 (36.3)	177 (63.7)			
4	14 (13.1)	93 (86.9)			<0.01**
1-3	266 (40.6)	389 (59.4)	1	(Reference)	
4	14 (13.1)	93 (86.9)	4.53	2.50-8.80	<0.01***
Hearing loss					
low frequencies	227 (34.8)	426 (65.2)	1.77	1.15-2.73	<0.01**
middle frequencies	258 (36.0)	459 (64.0)	1.70	0.88-3.26	0.11
high frequencies	234 (34.5)	444 (65.5)	2.29	1.42-3.74	<0.01**
Initial Grade of hearing loss, healthy ear					
0	181 (37.5)	302 (62.5)			
1	78 (41.7)	109 (58.3)			
2	15 (28.3)	38 (71.7)			
3	4 (17.4)	19 (82.6)			
4	2 (12.5)	14 (87.5)			0.02*

0-1	259 (38.7)	411 (61.3)	1	(Reference)	
2-4	21(22.8)	71 (77.2)	2.13	1.26-3.74	<0.01**
Received systemic steroid therapy	267 (37.3)	448 (62.7)	0.64	0.30-1.27	0.21
Intratympanic steroid therapy (initial or salvage)	19 (47.5)	21 (52.5)	0.63	0.31-1.26	0.18
Latency to begin steroid treatment[a]					
0-7 days post-onset	242 (40.2)	360 (59.8)	1	(Reference)	
8 or more days post-onset	38 (23.8)	122 (76.2)	2.16	1.43-3.31	<0.01***
Received prostaglandin E1 treatment	52 (39.7)	79 (60.3)	0.86	0.58-1.29	0.49
Received hyperbaric oxygen therapy	0(0)	12 (100)	Inf	1.64-Inf	<0.01**

[a]Some patients never received steroid treatment; they were manually assigned to the 8-or-more-days group.

SD, standard deviation; TIA, transient ischemic attack; PTA, pure tone audiogram; mean PTA, arithmetic mean of individual PTAs at 5 hearing frequencies (250, 500, 1000, 2000, 4000 Hz); OR, odds ratio; CI, confidence interval; MHLW, Ministry of Health, Labour, and Welfare of Japan (2015) criteria for diagnosing ISSNHL.

*p<0.05, ** p<0.01, ***p<0.001.

Supplemental Table 3 Sensitivity analysis using IPW-weighted multivariate logistic regression model

		Adjusted OR	95% CI	P-value
Age				
> 60		1	(Reference)	
≥ 60		1.32	0.92-1.89	0.13
Diabetes mellitus		0.83	0.53-1.30	0.43
Hypertension		0.60	0.41-0.88	<0.01**
Hyperlipidemia		0.84	0.56-1.25	0.38
Vertigo/dizziness		2.58	1.74-3.82	<0.01***
Initial MHLW grade of hearing loss in affected ear	1-3	1	(Reference)	
	4	3.39	1.82-6.28	<0.01***
Initial grade of hearing loss in healthy ear	0-1	1	(Reference)	
	2-4	1.89	1.09-3.29	0.02*
Hearing loss at low frequencies		1.33	0.82-2.14	0.25
Hearing loss at middle frequencies		1.71	0.86-3.43	0.13
Hearing loss at high frequencies		1.96	1.21-3.17	<0.01**
Regular anticoagulant medication at onset of hearing loss		2.91	1.07-7.95	0.04*
Systemic steroid therapy		1.18	0.54-2.58	0.67
Intratympanic steroid therapy (initial or salvage)		0.71	0.35-1.45	0.35

Latency to begin of steroid treatment	0-7 days	1	(Reference)	
	8 or more days	2.83	1.76-4.56	<0.01***
Prostaglandin E1		0.83	0.55-1.28	0.40

Dependent variable = no recovery.

OR, odds ratio; CI, confidence interval; MHLW, Ministry of Health, Labour, and Welfare of Japan (2015) criteria for diagnosing ISSNHL.

*p<0.05, ** p<0.01, ***p<0.001.
